# Mesenchymal stem cell-derived secretomes-enriched alginate/ extracellular matrix hydrogel patch accelerates skin wound healing

**DOI:** 10.1186/s40824-023-00446-y

**Published:** 2023-10-31

**Authors:** Jae Won Kwon, Cininta Savitri, Byoungha An, Seung Won Yang, Kwideok Park

**Affiliations:** 1 Center for Biomaterials, Korea Institute of Science and Technology (KIST), Seoul, 02792 Republic of Korea; 2 Division of Bio-Medical Science and Technology, KIST school, University of Science and Technology (UST), Seoul, 02792 Republic of Korea

**Keywords:** Alginate hydrogel, Cell-derived extracellular matrix (ECM), Human mesenchymal stem cells (hMSCs), Secretomes, Wound healing patch

## Abstract

**Background:**

The secretomes of mesenchymal stem cells (MSCs) have great therapeutic potential and thereby their efficient delivery into the target site is of particular interest. Here, we propose a new strategy of hMSCs-derived secretomes delivery for advanced wound healing upon harnessing the working principle of extracellular matrix (ECM)-growth factors interaction in vivo.

**Methods:**

We prepared an alginate hydrogel based wound patch, where it contains both human MSC-derived secretomes and ECM. The ECM was obtained from the decellularization of in vitro cultured human lung fibroblasts. The alginate solution was blended with ECM suspension, crosslinked, air-dried, then rehydrated with the secretomes contained in the concentrated conditioned media (CCM) as a highly saturated form of conditioned media (CM). We tested four different groups, with or without the ECM to investigate not only the role of ECM but the therapeutic effect of secretomes.

**Results:**

The secretomes reserved many, diverse bioactive factors, such as VEGF, HGF, IGFBPs, IL-6, and IL-8. Alginate/ECM/CCM (AEC) patch could hold significantly larger amount of secretomes and release them longer than the other groups. Our AEC patch was the most effective in stimulating not only cell migration and proliferation but the collagen synthesis of dermal fibroblasts in vitro. Moreover, the AEC patch-treated full-thickness skin wounds disclosed significantly better wound healing indications: cell recruitment, neovascularization, epidermis thickness, keratinocyte migration, and mature collagen deposition, as assessed via histology (H&E, Herovici staining) and immunofluorescence, respectively. In particular, our AEC patch enabled a phenotype shift of myofibroblast into fibroblast over time and led to mature blood vessel formation at 14 day.

**Conclusions:**

We believe that ECM certainly contributed to generate a secretomes-enriched milieu via ECM-secretomes interactions and thereby such secretomes could be delivered more efficiently, exerting significant therapeutic impact either individually or collectively during wound healing process.

**Graphical abstract:**

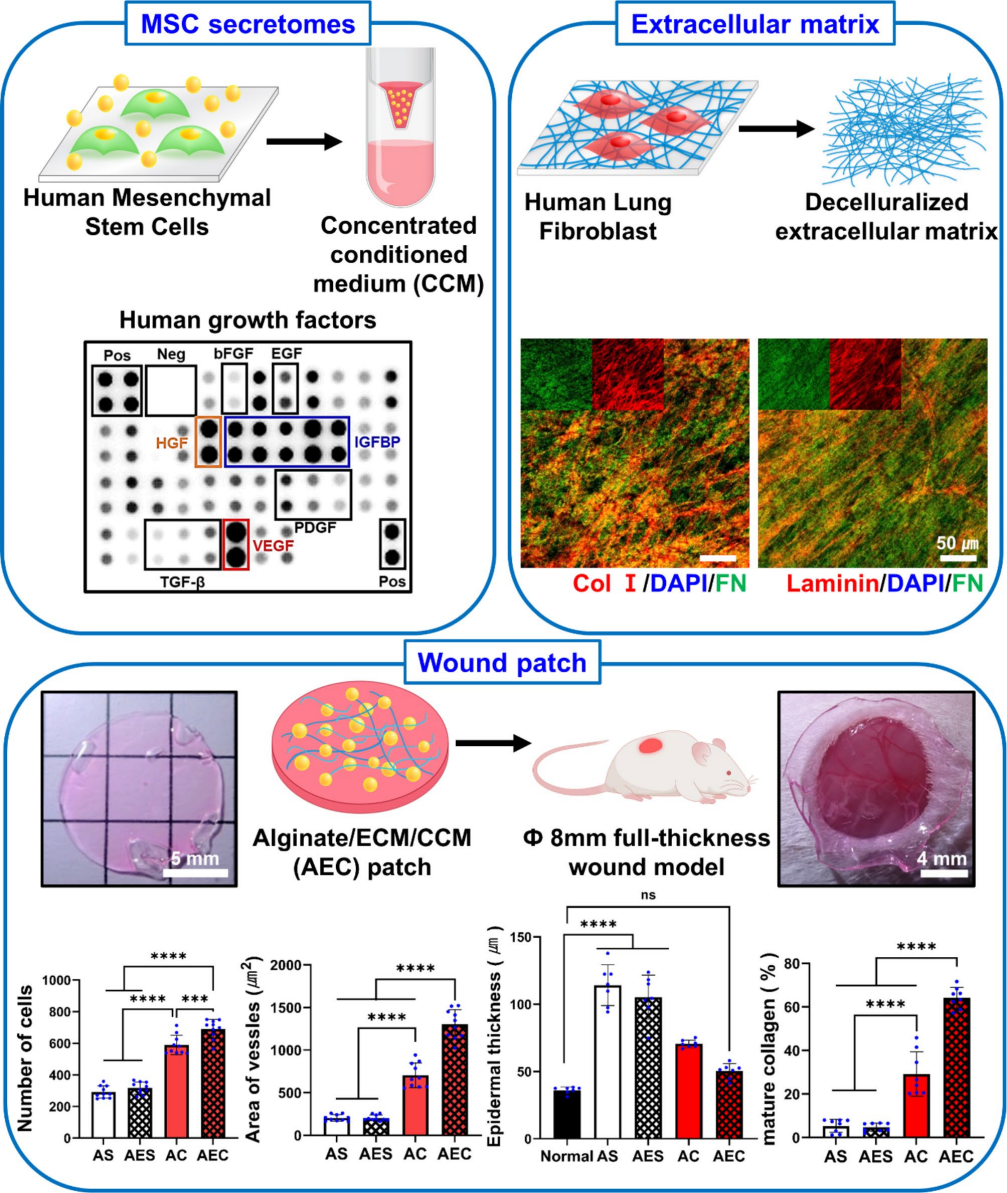

**Supplementary Information:**

The online version contains supplementary material available at 10.1186/s40824-023-00446-y.

## Background

The skin covers the entire body and has various functions, such as excretion, temperature regulation, sensation, and protection from external stimuli or harms. It is divided into three different layers, such as epidermis, dermis and hypodermis. The skin contains various appendages for proper functions, including sebaceous glands, hair follicles, blood vessels, lymphatic vessels, nerves, and muscles [1]. There is a natural healing mechanism of simple wounds but chronic ones are rarely self-healed, caused by various factors, such as aging, diabetes, burn, and cancer. [2]. Skin wound healing process undergoes a series of steps, including inflammation, proliferation, matrix formation, and remodeling, where they are tightly regulated by various factors and cells [3, 4]. Although many attempts have been made for effective healing using wound dressings, they have showed therapeutic limitations, and thus should be improved for more advanced wound regeneration [5].

Mesenchymal stem cells (MSCs) are multipotent stem cells obtained from diverse adult tissues, such as adipose tissue, bone marrow, and umbilical cord. MSC differentiate into different cell types and they are a primary source of cell therapy in clinic [6–8]. As an alternative to cell transplantation, MSCs-derived secretomes have also been evaluated in many studies for their therapeutic potential [9–11]. The secretomes are the secreted factors from cells and they would reserve a variety of cytokines, chemokines, exosomes and growth factors [12, 13]. In fact, the MSCs secretomes have proven the therapeutic effects in the wound healing [14, 15]. However, more advanced, biomaterial-based carrier system, where it can hold larger amount of secretomes and deliver them more efficiently is still required for better wound regeneration [16, 17]. In fact, the secretomes serve as the important mediators in the wound healing process by stimulating and orchestrating the contributing cells, such as keratinocytes, fibroblasts, and endothelial cells. For example, epidermal growth factor (EGF), fibroblast growth factor (FGF), transforming growth factor-β (TGF-β), interleukin-1 (IL-1), interleukin-6 (IL-6), and tumor necrosis factor-α (TNF-α) are crucial for re-epithelialization. Vascular endothelial growth factor (VEGF), hepatocyte growth factor (HGF), FGF, and platelet-derived growth factor (PDGF) play an important role in angiogenesis and granulation tissue formation. Moreover, FGF and PDGF are deeply involved in matrix formation and remodeling [18, 19].

Alginate is a natural polymer obtained from the seaweed and has been widely used in foods, pharmaceuticals, fertilizers, cosmetics, and medicine. Alginate has been harnessed as an attractive wound dressing material because it is biocompatible, hydrophilic, easy to handle, and durable [20, 21]. Alginate hydrogel dressing is commercially available, providing key functions, such as moist environment, wound exudate absorption, and swelling [22, 23]. On the other hand, the extracellular matrix (ECM) is a complex of diverse macromolecules, i.e., fibronectin, collagens, laminin, and proteoglycans as a major component. It can regulate many cellular functions, including cell adhesion, proliferation, migration, and differentiation. Among the multiple functions of ECM, we particularly paid attention to the growth factors (GF) binding capacity of the ECM. For example, fibronectin binds to VEGF, FGF, and HGF [24, 25]. Laminin also binds to VEGF, PDGF, insulin-like growth factor (IGF) and bone morphogenetic protein (BMP) [26]. To this end, we selected biomimetic, natural ECM, i.e., cell-derived, decellularized ECM as a candidate material of bioactive secretomes delivery, because our ECM holds diverse ECM constituents, including fibronectin and laminin, where they reserve intrinsic capability of GFs binding.

**Scheme 1:**
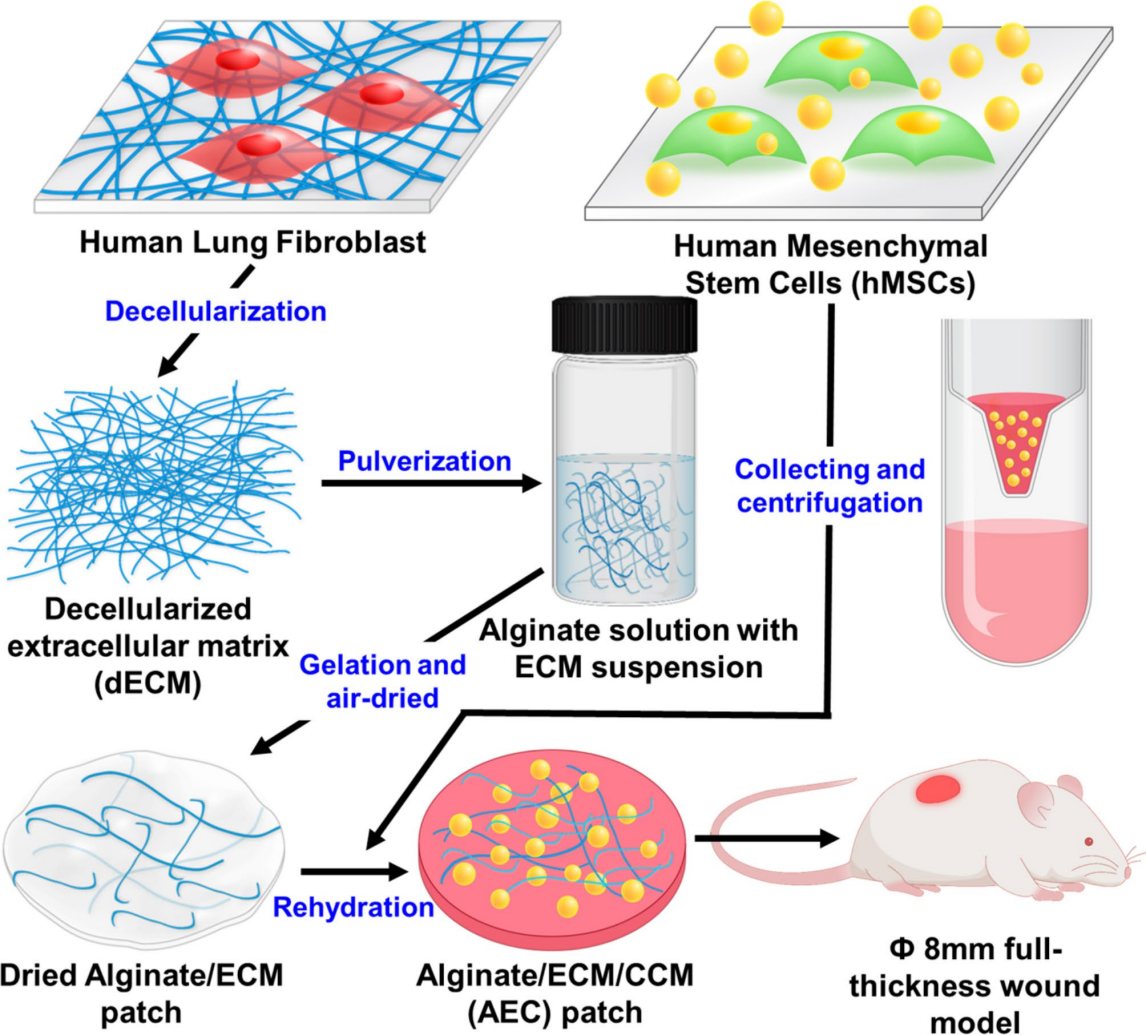
Schematic of the whole fabrication process in preparing alginate/ECM/CCM (AEC) patch. The decellularized ECM, ECM was suspended homogenously via ultrasonication, then mixed with 5% sodium alginate solution, subsequently followed by gelation of sodium alginate using 500mM CaCl_2_ solution. Meanwhile, concentrated conditioned medium (CCM) was prepared by centrifugation of the conditioned medium of hMSC. Finally, AEC patch was produced by rehydration of the dried alginate/ECM patch using the CCM. Our AEC patch was transplanted in the 8 mm full-thickness wound and extensively assessed for wound healing capability

In this study, we propose a new alginate patch, where it contains MSC-derived secretomes as well as human fibroblast-derived, decellularized ECM. We hypothesize that ECM containing alginate patch can effectively hold and enable a sustained release of secretomes, presumably through the growth factors binding capability in the ECM, eventually leading to significantly advanced therapeutic effect. Our primary interest was to secure our patch with a large amount of secretomes through the ECM and thereby to deliver those therapeutic biomolecules more efficiently into the skin wounds. To this end, concentrated conditioned medium (CCM) was prepared by centrifugation, where it contained highly enriched secretomes and alginate/ECM patch was then rehydrated with the CCM, eventually yielding alginate/ECM/CCM (AEC) patch (Scheme 1). Along with extensive in vitro and in vivo experiments, we confirmed that our AEC patch could not only protect the wound site, providing a moist environment as wound dressing, but accelerate the wound healing process through a pro-healing mechanism, primarily triggered by sustained delivery of enriched growth factors/cytokines in the secretomes.

## Materials and methods

### Preparation of fibroblast-derived, decellularized ECM

Human lung fibroblasts (WI-38, CCL-75; ATCC) were cultivated on a tissue culture plate (TCP) at a density of 2 × 10^4^ cells/cm^2^ in Dulbecco’s modified Eagle’s medium (DMEM) supplemented with 10% fetal bovine serum (FBS), 100 U/mL penicillin and 100 µg/mL streptomycin (1% P/S) under a normal culture condition (5% CO_2_, 37℃). The medium was replenished every 2–3 day. On day 10, those confluent cells were rinsed twice with phosphate buffered saline (PBS) and subjected to decellularization via dispensing a solution of 0.25% Triton-X 100 and 20mM NH_4_OH into the plates, followed by PBS washing and subsequent treatment with 50 U/mL DNase I (18,047 − 019; Invitrogen) and 100 µL /mL RNase A (12,091 − 039; Invitrogen) for 2 h at 37℃. After several washing with PBS, the decellularized extracellular matrix ECM was transferred in a conical tube and kept at -20℃ in deionized water (DW) for further use. The entire process was performed in a sterile condition.

### Preparation of hMSC-derived secretomes in concentrated conditioned medium

Human bone marrow-derived mesenchymal stem cells (hMSC; PT-2501, Lonza) were cultivated on the TCP at the density of 2 × 10^4^ cells/cm^2^ in DMEM containing 10% FBS and 1% P/S. At 80% confluence, those cells were washed with PBS and incubated for 24 h in fresh serum-free medium (SFM) at 37℃. The medium was collected and centrifuged at 1,000 rpm for 5 min to eliminate the cell debris. Next, the concentrated conditioned media (CCM) of the hMSC-derived secretomes was then obtained via another centrifugation at 3,600 rpm for 30 min at 4℃ using an Amicon Ultra-15 Centrifugal Filter Unit with Ultracel-3 membrane 3 kDa molecular weight cut-off (UFC900324, Merck Millipore Ltd., Burlington, MA, USA). The supernatant inside the filter units were carefully collected and stored at -20℃ until further use. The whole process was carried out in a sterile condition. The resulting CCM was profiled using human growth factors microarray and human cytokine array as described in 2.4.

### Immunofluorescence (IF) staining

The samples were fixed with 4% paraformaldehyde for 15 min at room temperature (RT) and then rinsed three times with PBS. They were permeabilized with 0.2% Triton-X 100 for 10 min, washed with PBS, and blocked with 3% bovine serum albumin (BSA) for 1 h at RT. They were then incubated with the primary antibodies overnight at 4℃, rinsed with PBS, then incubated with the secondary antibodies for 1 h at RT. After several washes, those samples were mounted onto microscope glass slides using Vectashield® mounting medium containing 4, 6-diamidino-2-phenylindole (DAPI) (H1200; Vector Lab, Burlingame, CA, USA) for the nuclei labeling. Fluorescence images were taken using a confocal laser scanning microscope (LSM 700; Carl Zeiss, Oberkochen, Germany). The primary antibodies and dilution ratios are as follows: rabbit polyclonal anti-collagen type I (ab34710; Abcam, Cambridge, UK, 1:300), mouse monoclonal anti-fibronectin (SC-8422; Santa Cruz Biotechnology, 1:200), rabbit polyclonal anti-collagen type III (ab7778; Abcam, 1:300), rabbit polyclonal anti-laminin (L9393, Sigma-Aldrich, 1:100) and rabbit polyclonal anti-CD31 antibody (ab28364; Abcam, 1:200). The secondary antibodies are Alexa Fluor® 488-conjugated goat anti-rabbit IgG (A-11,008; Invitrogen, Waltham, MA, USA) and Alexa Fluor® 594-conjugated donkey anti-rabbit IgG (A-21,207; Invitrogen), both of which were diluted at 1:300. The F-actin cytoskeleton of HUVEC was observed using ActinRed™ 555 ReadyProbes™ Reagent, an F-actin probe (phalloidin) conjugated to the red-orange fluorescent dye tetramethylrhodamine (TRITC) (R37112; Invitrogen, 1:200).

### Profiling of the secretomes in the CCM

To elucidate the secretome components contained in the CCM, the Human Growth Factor Antibody Array Membrane (41 targets, ab134002; Abcam) was employed according to the manufacturer’s instructions. Briefly, once the membrane was blocked with a blocking buffer at RT, it was then incubated with the CCM overnight at 4℃ on a shaker. After washing with a washing buffer, the membrane was incubated with biotin-conjugated anti-cytokines overnight at 4℃ with agitation, followed by another overnight incubation with HRP-conjugated streptavidin at 4℃. The membrane was extensively washed and finally added with a detection buffer. Moreover, the cytokines in the CCM were also examined using the Proteome Profiler™ human cytokines array kit (ARY005B; R&D systems). In brief, the nitrocellulose membrane was blocked with a block buffer for 1 h at RT. Subsequently, a mixture of the CCM and biotinylated detection antibodies was added onto the membrane and then incubated overnight at 4℃ on a shaker. After several washes, the membranes were incubated for 30 min with streptavidin-horseradish and then treated with chemiluminescence detection reagents. Chemiluminescence blot detection was carried out via iBright CL1500 imaging system (Invitrogen, Waltham, MA, USA). The collected data were quantitatively analyzed using iBright analysis software 4.0.1. The results are presented as the relative ratio (%) between the positive dots and the reference dots.

### Fabrication of alginate hydrogel patches

Sodium alginate powder (9005-38-3, 500–600 cP; FUJIFILM Wako Chemicals, Osaka, Japan) was purchased and sterilized under UV light overnight before solubilization in autoclaved DW. Alginate solution (3 or 5%, w/v) was produced by continuous stirring using a magnetic bar at 100℃ overnight. Meanwhile, the ECM suspension was prepared in DW via pulverization using an ultrasonicator (Sonic Dismembrator Model 500; Thermo Fisher Scientific, Waltham, MA, USA). Total proteins content in the ECM suspension was quantitatively measured via BCA protein assay (23,225, Thermo Fisher Scientific). The resulting ECM suspension was then homogeneously mixed with 5% alginate solution and stirred 1 h to yield an alginate/ECM solution, where it contains both alginate (3%, final conc.) and ECM (0.1%, w/v). Subsequently, 100 µL of alginate/ECM solution was poured into 12-well plate, respectively and pressed with an 18 mm diameter coverslip to produce a thin membrane. The alginate hydrogel membrane was then crosslinked by dispensing 2 mL of 500 mM calcium chloride at 37℃. The resulting membrane was peeled off from coverslip, washed three times with DW, punched in 12 mm diameter using biopsy punch, and air-dried in a clean bench. To include hMSC-derived CCM, the dried alginate patch was rehydrated with 120 µL of CCM or SFM (a negative control) at 37℃. The overall fabrication process was illustrated in the Scheme 1. Moreover, alginate hydrogel patches were prepared with or without ECM, where they were also added with CCM or SFM. As a result, there are four experimental groups in this study: alginate/SFM (AS), alginate/ECM/SFM (AES), alginate/CCM (AC), and alginate/ECM/CCM (AEC).

### Scanning electron microscope observation

To confirm the surface morphology of alginate and alginate/ECM patch, the lyophilized samples were placed on carbon tape, then platinum-coated for 1 min using ion sputter (E-1045, Hitachi, Tokyo, Japan), and observed using scanning electron microscope (SEM; Phenom Pro G6 Desktop SEM, Thermo fisher scientific, Massachusetts, USA).

### Characterization of AEC hydrogel patches

We examined the physical and mechanical property of alginate hydrogel patches, particularly paying attention to the difference between AC and AEC hydrogel. The test samples were prepared as a patch type as described above for water contact angles measurement. A contact angle measurement device (Attension Theta flow, Biolin Scientific, Sweden) equipped with a video camera was employed to measure the hydrophilicity and wettability of each patch. Static sessile drop method was performed with a droplet size of 5.0 µL and contact angles were measured immediately after the drop, where they were calculated using Young-Lapace equation. The rheological property of AC and AEC hydrogel sample (10 mm diameter, 1 mm thick) was also examined via an Anton Paar Rheometer (MCR102; Anton Paar, Austria). The rheometer was equipped with a parallel plate (25 mm dia.) and the sample gap size was 0.35 mm. We determined both storage modulus (G’) and loss modulus (G”) while applying 3% shear strain at RT. The collected data were analyzed using RheoCompass software. For the mechanical property, cylindrical patch samples (5 mm diameter, 3 mm height; n = 3, each group) were prepared and tested using Universal Testing machine (Instron® 5566, Instron Corporation, USA), where they were compressed to 60% of the whole thickness under 1.0 mm/min speed. We acquired stress-strain curves and calculated the compressive strength using Bluehill® 2 software.

### Swelling property and biodegradation

We prepared alginate and alginate/ECM patch, air-dried them, then weighed (*W*_*0*_). These samples were then immersed in normal saline for 30 min, 1, 2, 3, 4, and 5 h at 37℃. Once we removed the surface water, they were weighed at each time point (*W*_*t*_). Each sample was repeated five times. The swelling ratio of test sample (n = 5, each group) was calculated using this formula:

$${\text{Swellingratio}} = \frac{{{W_t} - {W_0}}}{{{W_0}}} \times 100\%$$

For biodegradability analysis, they were dipped in normal saline (pH 6.5) added with 2 mg/mL lysozyme (10,837,059,001; Roche, Basel, Switzerland) and incubated at 37℃, where the medium was replaced every other day. Those patch samples were collected at 1, 2, 3, 4, 5, 6, and 7 day (*W*_*t*_), respectively, then lyophilized and weighed. As a control, the same patch samples were incubated for 24 h at 37℃ in normal saline without lysozyme, lyophilized and weighed (*W*_*0*_). Each experiment was conducted in triplicates. The degradation ratio was calculated using this formula:

$${\text{Degradationratio}} = 100\% - \left( {\frac{{{{\text{W}}_{\text{t}}}{\text{ - }}{{\text{W}}_{\text{0}}}}}{{{{\text{W}}_{\text{0}}}}} \times {\text{100\% }}} \right)$$

### Release profile of secretomes from alginate patches

To investigate the release profile of secretomes out of either AC or AEC patch, the release rate and amount was evaluated with time via BCA protein assay. Each patch sample was put in the 500 µL of DW and remained at 37℃ for 96 h. During the time period, we collected 100 µL supernatant at specific time points (2, 4, 8, 12, 24, 48, 72, 96 h) and subsequently replenished 100 µL of fresh DW. For each AC and AEC group, five patches were prepared, respectively and examined in triplicates at each time points. In addition, we opted a specific time point, 72 h and thoroughly evaluated the total amount of released secretomes for 72 h using the collected medium (ten patch samples for each group). The assay was performed in accordance with the manufacturer’s instructions. Quantitative analysis was carried out based on the standard curve. Meanwhile, to determine amount of GFs contained in the AC or AEC patch, each sample was incubated in DW at 37℃ for 3 days. We collected the supernatant and measured the amount of each GF using enzyme-linked immunosorbent assay (ELISA) kit, including human hepatocyte growth factor (HGF; DY294), human Insulin-like growth factor binding protein 1 (IGFBP-1; DY871) and human vascular endothelial growth factor (VEGF) (DVE00, R&D Systems, MN, USA) according to the manufacturer’s protocols. Moreover, the cytokines released from the two patches were also evaluated via IL-6 (DY206) and Interleukin-8 (IL-8) ELISA kits (DY208, R&D Systems). Each group was tested in triplicates from different samples.

### Cell scratch assay and cell proliferation test

Human skin fibroblasts (hSFB; BJ, ATCC) were seeded and cultivated on the 12-well plate at the density of 2 × 10^4^ cells/cm^2^ in DMEM containing 10% FBS and 1% P/S until confluence. A wound scratch was created by scraping the center of plate using a 200 µL pipette tip in a straight line. After a brief wash with PBS, the medium was replaced with SFM (DMEM with 1% P/S). Meanwhile, we also prepared transwell inserts with 8.0 μm pore size (353,097; FALCON), in which four different types of alginate patches were loaded (1 patch/ 1 insert), respectively and being allowed to release the contents included in each patch system. Each group was tested in triplicates. The wound area was closely monitored at specific time points (0, 6, 12, 18, and 24 h) using optical microscopy. The remaining wound area was quantitatively calculated using Image J as a ratio between the wound area at each time point and the one at 0 h. In addition, to assess the proliferation of hSFBs under the same transwell setting, those cells were seeded and cultivated for 24 h. After replacing the medium to SFM, we placed those alginate patches on the transwell insert and allowed the release of the contents out of the patches. Cell proliferation was examined at 3, 7, and 10 day (*n = 3*, each group) using the cell counting kit-8 (CCK-8) assay (CK04; Dojindo, Kumamoto, Japan). The results were quantitatively presented, based on the standard curve of known number of hSFB.

### Analysis of collagen deposition and HUVEC assembly in vitro

To evaluate collagen deposition by skin fibroblasts, when treated with the released secretomes, hSFB were seeded on the gelatin-coated coverslip in 12-well plate under the same condition as described in 2.8. Those cells were then washed with PBS and the medium was changed to SFM. Four different alginate patches were located on top of the hSFB using transwell inserts and remained for 3 days (1 patch/ 1 insert). After then, they were fixed and prepared for IF staining of collagen type 1 (Col 1). The samples were tested in triplicates for each group. Collagen deposition by hSFB was quantitatively determined using Image J by measuring the Col 1-positive area (five random images, each group). On the other hand, human umbilical vein endothelial cells (HUVECs) (C2517A; Lonza, Basel, Switzerland) were seeded on gelatin-coated cover glass at the density of 2 × 10^4^ cells/cm^2^ and cultivated in endothelial cell growth medium (EGM-2 BulletKit, CC-3162; EBM-2 with supplements kit, Lonza). After cultivating HUVECs for 24 h, we washed them with PBS and changed the medium to endothelial cell basal medium (EBM-2, CC-3156; without supplements kit, Lonza). Likewise the collagen deposition test protocols, we followed the same procedure except the use of HUVECs. After 48 h, the cells were fixed and assessed via F-actin and CD31 IF staining. A self-assembly of HUVECs was scrutinized and quantified using image J software, based on the F-actin positive area appeared in the IF images (five random images, each group).

### Keratinocyte migration assay in vitro

Human epidermal keratinocyte cells (CB-HK-001; CEFOBio, Gyeonggido, Korea) were cultured in keratinocyte growth medium 2 kit (KGM2, C-20,011, PromoCell, Germany), supplemented with 1% P/S. To investigate the effect of released secretomes on cell migration, firstly, four different alginate patches were incubated (1 patch/ 1 well) for 24 h in KBM2 with 20% KGM2 SupplementMix. Then, the keratinocytes suspended in keratinocyte basal medium 2 (KBM2, C-20,211, PromoCell) without supplement were seeded in the transwell insert with 8 μm pore size (353,097, FALCON) at the density of 1 × 10^5^ cells (Fig. S2A). After 24 h incubation, once the cells in the upper transwell membrane was cleared with cotton swab, only the keratinocytes on the lower surface were fixed with 100% of cold methanol for 10 min at 4℃. The fixed keratinocytes were stained with 1% crystal violet solution (61,135, Sigma-Aldrich, St. Louis, MO, USA) for 20 min at RT, then subjected to air-dry, and confirmation of the migrated keratinocytes using optical microscope. For each insert, three different fields were randomly selected and the number of keratinocytes were counted. This experiment was conducted in triplicates.

### Murine full-thickness skin wound model

Therapeutic effects of the secretomes were further investigated using full-thickness skin wound model. For this, the male BALB/c mice (6-week-old) were purchased form Orient Bio (Gapyeong, Korea). They were randomly divided into four groups according to the treatment regimens: AS, AES, AC, and AEC (*n* = 3 per group). Before surgery, they were anesthetized by gas inhalation using isoflurane in oxygen. Mouse hair was shaved, and the dorsal skin was then scrubbed using alcohol gauze for sterilization. Full-thickness wounds were created by using a biopsy punch (8 mm) under sterile surgical condition. Each patch, prepared as described in 2.5, was placed on the wound site (two wounds per mouse). Subsequently, Tegaderm™ Film and Coban bandage were wrapped around the wounds to keep the patches from detachment and to protect the treatment. These patches and dressing films were replaced every 2–3 day. The gross observation of wound closure was carried out on day 0, 2, 4, 7 and 14 post-treatments. The remaining wound area at specific time point (four random images, each group) was quantitatively calculated to assess wound closure rate using Image J software as a percentage of the wound region normalized to that of day 0. The animal experiments we carried out comply with the National Research Council’s Guide for the Care and Use of Laboratory Animals (8th edition, NIH Publication, 2011). All the animal studies were also approved by the Korea Institute of Science and Technology Animal Care and Use Committee (KIST-IACUC-2022-05-083).

### Subcutaneous transplantation for in vivo safety

For investigation of in vivo safety of our AEC patch, the male BALB/c mice (6-week-old) obtained from Orient Bio (Gapyeong, Korea). They were anesthetized by gas inhalation using isoflurane in oxygen before surgery. After hair shaved, the skin was wiped with alcohol swab and scrubbed using gauze with povidone iodine. Minimal incision was created using scissors and AEC patches were transplanted in subcutaneous. Two AEC patches were transplanted each mouse (n = 2) on two different back parts. The incised area was sutured and covered with Tegaderm^™^ Film. The mice were euthanized by using CO_2_ inhalation at day 3. And the remained AEC patch was collected with around tissue. Behavioral abnormalities or death of mice were checked, and inflammatory reactions or adverse reactions were confirmed through histological staining.

### Histological analysis of wound tissues

On day 7 and 14, the mice were euthanized by using CO_2_ inhalation, then followed by the excision of the skin wound tissues. Such tissue samples were fixed with 10% formalin, embedded in paraffin blocks, and sectioned in 5 μm thickness across the tissues. These thin sections were deparaffinized using xylene and rehydrated in a series of alcohol solutions. They were then subjected to hematoxylin and eosin (H&E) staining to evaluate the degree of skin tissue regeneration, where four different alginate patches were administered, respectively. Collagen deposition and the degree of maturity were also examined via Herovici (KTHERPT; StatLab, Mckinney, TX, USA) staining. Based on the randomly selected, high resolution images (8–10, each group) as obtained using an inverted microscope (Axio Vert.A1; Carl Zeiss, Oberkochen, Germany), we quantitatively assessed cell recruitment and neovascularization at 7 day as well as epidermal thickness and mature collagen deposition at 14 day using Image J.

### Immunohistochemical analysis

To further understand the wound healing efficacy of four different treatment regimens via immunohistochemistry, the tissue sections were deparaffinized, rehydrated, then peroxidase blocked, and followed by antigen retrieval using microwave heating in citrate buffer (pH 6). After the blocking with 1% BSA, we incubated them with primary antibodies at 4℃ overnight. They were then rinsed with PBS several times and subsequently treated with secondary antibodies for 1 h at RT. Finally, those samples were counter-stained with NucBlue™ Live ReadyProbes™ Reagent (R37605, Invtirogen) and mounted using VECTASHIELD® Antifade Mounting Medium (H-1000; Vector Lab). The primary antibodies we used are as follows: rabbit monoclonal anti-cytokeratin 10 (ab76318; Abcam, 1:500), mouse monoclonal alpha smooth muscle actin (α-SMA) (A2547; Sigma-Aldrich, 1:400) and rabbit polyclonal anti-CD31 antibody (ab28364; Abcam, 1:100), mouse monoclonal vimentin antibody (E-5) (SC-373,717; Santa Cruz Biotechnology, Dallas, TX, USA) and rabbit polyclonal anti-alpha smooth muscle actin (ab5694; Abcam, 1:100). The secondary antibodies are as follows: Alexa Fluor® 488-conjugated goat anti-rabbit IgG (A-11,008; Invitrogen), Alexa Fluor® 594-conjugated goat anti-mouse IgG (A-11,005; Invitrogen), Alexa Fluor® 488-conjugated goat anti-mouse IgG (A-11,001; Invitrogen) and Alexa Fluor® 594-conjugated donkey anti-rabbit IgG (A-21,207; Invitrogen). All secondary antibodies were diluted at 1:200. The stained samples were observed using confocal laser scanning microscope (Carl Zeiss). Based on the positive area as shown in the high-power field images (3–5 random ones, each group), we attempted to quantitatively assess various wound healing parameters, such as keratinocyte migration, mature blood vessel formation, and activated myofibroblasts using image J.

### Statistics

All the data are presented as mean ± standard deviation, along with individual data points. Statistical analysis was performed using a two-tailed (α = 0.05) Student’s t-test for the two experimental groups. One-way analysis of variance (ANOVA) with a post-hoc Tukey’s multiple comparison test was also carried out for more than three test groups. Two-way ANOVA with a post-hoc Tukey’s multiple comparison test was performed for more than three test groups with two or more variables. Statistically significant differences are denoted by *p < 0.05, **p < 0.01, ***p < 0.001, or ****p < 0.0001.

## Results

### Characterization of ECM and hMSC-derived secretomes

**Fig. 1:**
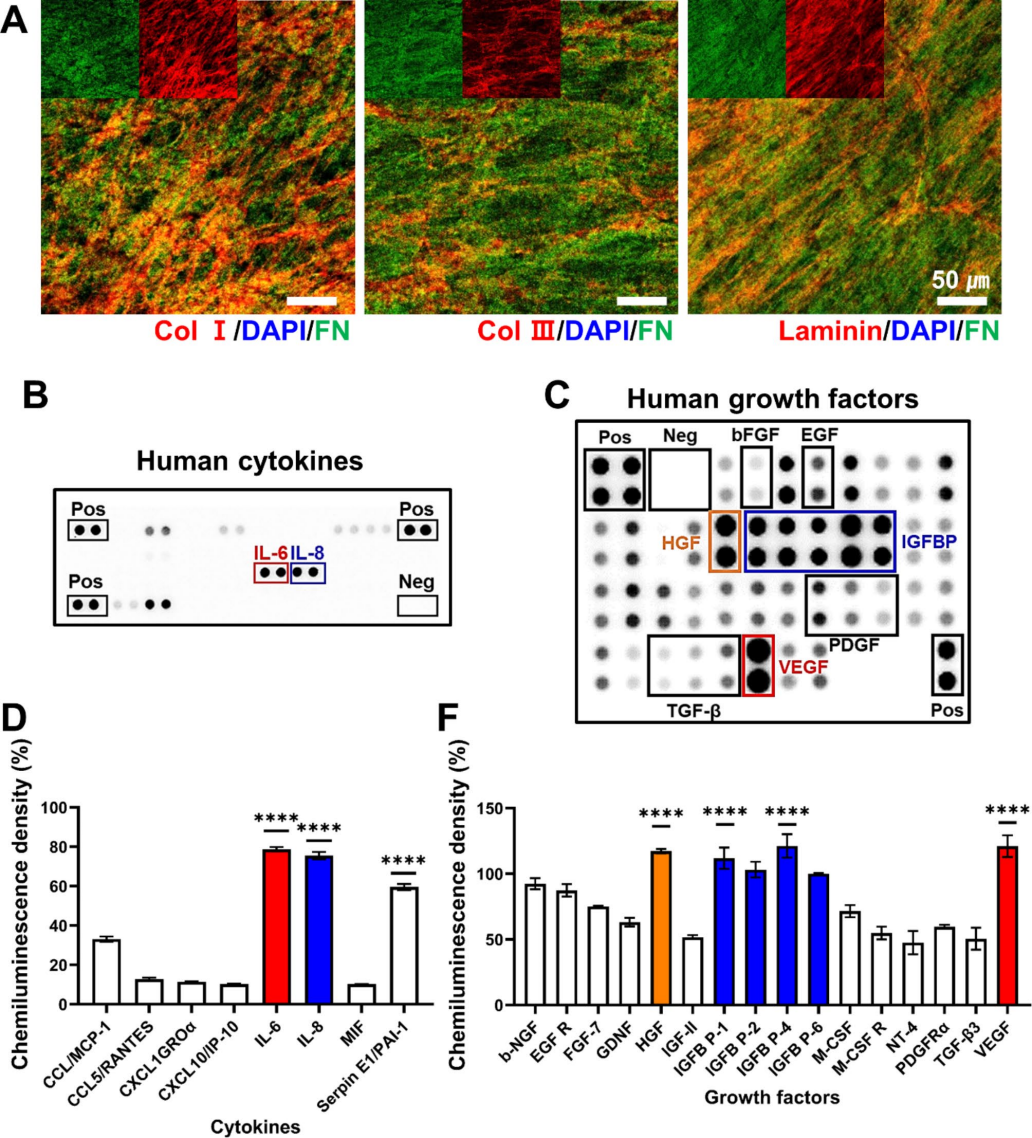
Characterization of ECM and CCM components. (**A**) Immunofluorescence staining of major ECM proteins (blue: DAPI, green: fibronectin, red: collagen type I, collagen III, laminin). (**B**) Analysis of CCM for human cytokines using proteomic microarray kit. (**C**) Antibody array of human growth factors in the CCM. Those positive signals (**B**) and (**C**) were normalized via the dot intensity of positive control (100%). (**D**) The cytokines detected with a normalized intensity (> 5%) were appeared in the graph (****p < 0.0001). (**E**) Positive dots of growth factors with a normalized intensity (> 20%) were quantitatively marked in the graph (**** *p* < 0.0001)

The human fibroblast-derived, decellularized ECM was examined through the detection of major ECM components, where collagen I, collagen III, laminin and fibronectin were identified using IF (Fig. 1A). DAPI (blue) staining showed the absence of nucleic material confirming a successful decellularization. Additionally, when we compared the relative amount of individual ECM using fibronectin (green) as a control, both collagen I (red) and laminin (red) were relatively abundant, followed by small amount of collagen III (red). On the other hand, CCM profiling disclosed a variety of cytokines and GFs as assessed via the human cytokine array and human growth factor array, respectively (Fig. 1B C). When the chemiluminescence intensity of those positive dots was evaluated, we noticed that out of the 36 cytokines detected, three cytokines were particularly notable: IL-6, IL-8 and Serpin E1/PAI-1 (Fig. 1D). Moreover, when we screened the GFs in the CCM, with the intensity of more than 50%, VEGF, HGF, and IGFBP levels were remarkably higher than the others (Fig. 1E).

### Fabrication and characterization of alginate hydrogel patch

**Fig. 2:**
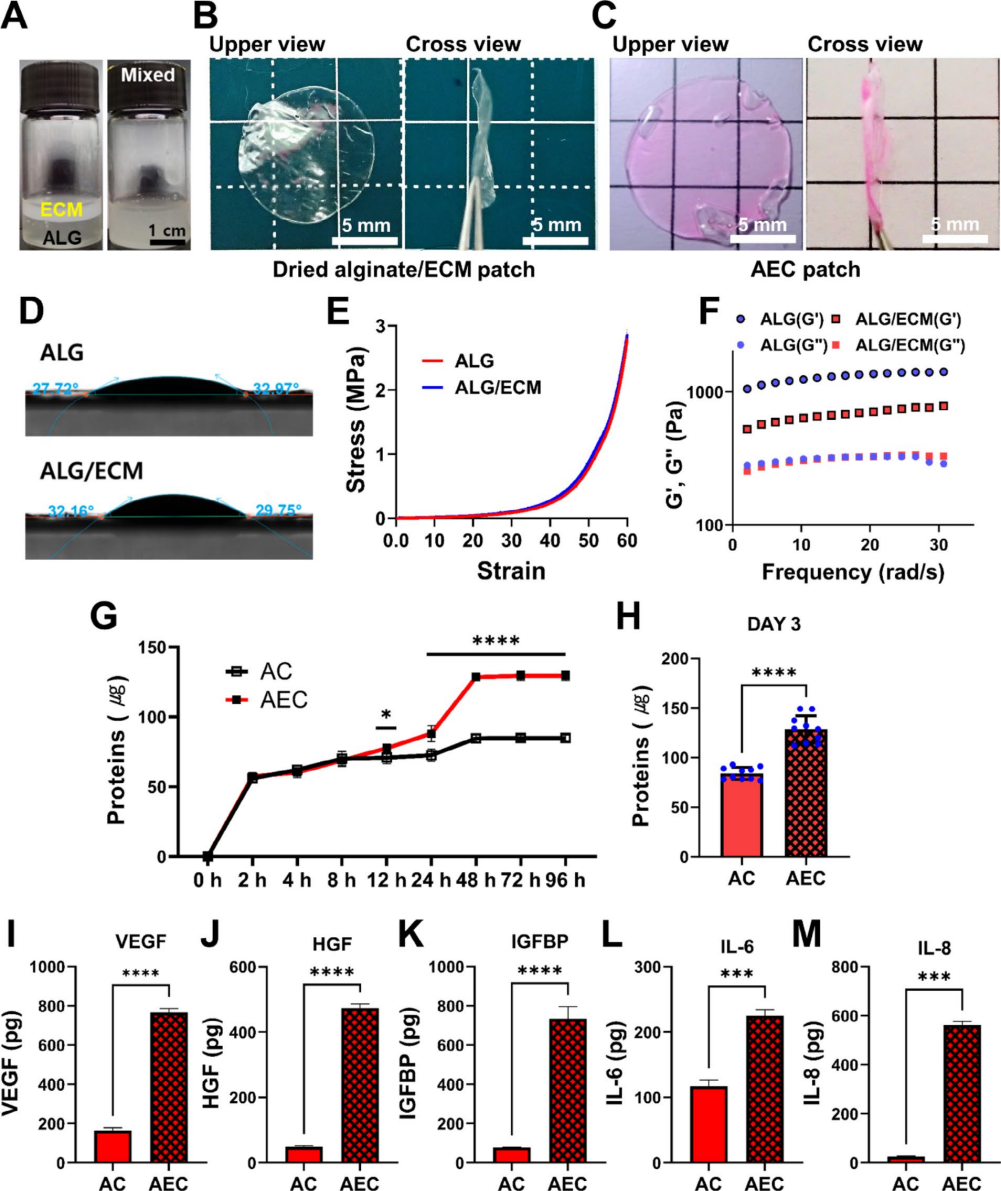
Preparation of AEC patch and their characterization. (**A**) Alginate solution before and after being mixed with ECM solution. (**B**) Optical image of dried alginate/ECM patch in upper and cross view. (**C**) The rehydrated AEC patch with CCM in upper and cross view. Scale bar is 5 mm. (**D**) Representative images of water contact angles for alginate (ALG) and alginate/ECM (ALG/ECM) patch. (**E**) A compressive stress-strain curve of alginate and ALG/ECM hydrogel. (**F**) Measurement of both storage (G’) and loss (G”) modulus in the ALG/ECM hydrogel. (**G**) Release profile of CCM as observed using AC and AEC patch, respectively for up to 96 h. (**H**) Total proteins content as assessed by the released proteins out of AC and AEC patch, respectively at 3 day. Quantitative analysis of released growth factors/cytokines out of AC and AEC patch: (**I**) VEGF, (**J**) HGF, (K) IGFBP, (**L**) IL-6 and (**M**) IL-8, respectively. Statistically significant difference (*p < 0.05, ***p < 0.001, ****p < 0.0001)

To fabricate AEC patch, we first prepared alginate (ALG)/ECM patch, where ALG solution and ECM suspension were homogeneously mixed (Fig. 2A), then cross-linked by using CaCl_2_, and air-dried (Fig. 2B). Both upper and cross view were presented, respectively. In SEM images, the surface topography of patches was smooth and they exhibited no significant difference (Fig. S1A). This ALG/ECM film was eventually rehydrated with the CCM, producing AEC patch (Fig. 2C), where the swelling ratio was estimated to about 1000% within 1 hr at 37℃ condition. In normal saline condition, that of ALG/ECM patch was 2464.2 ± 60.7%, slightly lower than that of ALG (2791.2 ± 65.2%) at 5 hr (Fig. S1B). The water contact angles measurement showed 27.75°~32.97° (ALG) and 29.75°~32.16° (ALG/ECM) (Fig. 2D), both of which were very similar to each other, suggesting a very hydrophilic surface property (θ < 90°). From the compression test, we noticed that both patches were still durable at 3 MPa without failure in the stress-strain curve (Fig. 2E). Both curves showed the same pattern, indicating that there is no effect of ECM on the mechanical property. Moreover, when the rheological property was investigated, both hydrogels (ALG, ALG/ECM) exhibited a viscoelastic property of hydrogel (G” < G’). Interestingly, the loss modulus (G”) was similar to each other but the storage modulus (G’) was quite different, where ALG had significantly higher G’ than that of ALG/ECM (Fig. 2F). In addition, when the degradation ratio was assessed, both patch types exhibited similar in vitro degradation pattern, where they began to degrade on day 4 and the degradation ratio (%) dropped to about 80% at 7 day (Fig. S1C).

### Release profile and loading efficacy of secretomes

To understand the release rate and loading efficiency, the secretomes contained in either AC or AEC patch were quantitatively measured in total protein levels. Both patches showed a similar release pattern at early time point, but the AEC patch released significantly more proteins after 12 h (Fig. 2G). The actual amount of secretomes released from the AEC patch at 3 day disclosed greatly higher contents over the AC patch (Fig. 2H). The difference was statistically significant. Upon the profile of GFs and cytokines release, we further investigated the individual GF release out of AEC and AC patch via ELISA. As a result, we learned that the concentrations of VEGF, HGF, and IGFBP released from the AEC patch were significantly larger than those of AC (Fig. 2I J, 2 K). Likewise, much higher amount of IL-6 and IL-8 was also detected from AEC patch over AC one (Fig. 2L M). They all showed statistically significant differences between them.

### Effect of secretomes from AEC patch in vitro

**Fig. 3:**
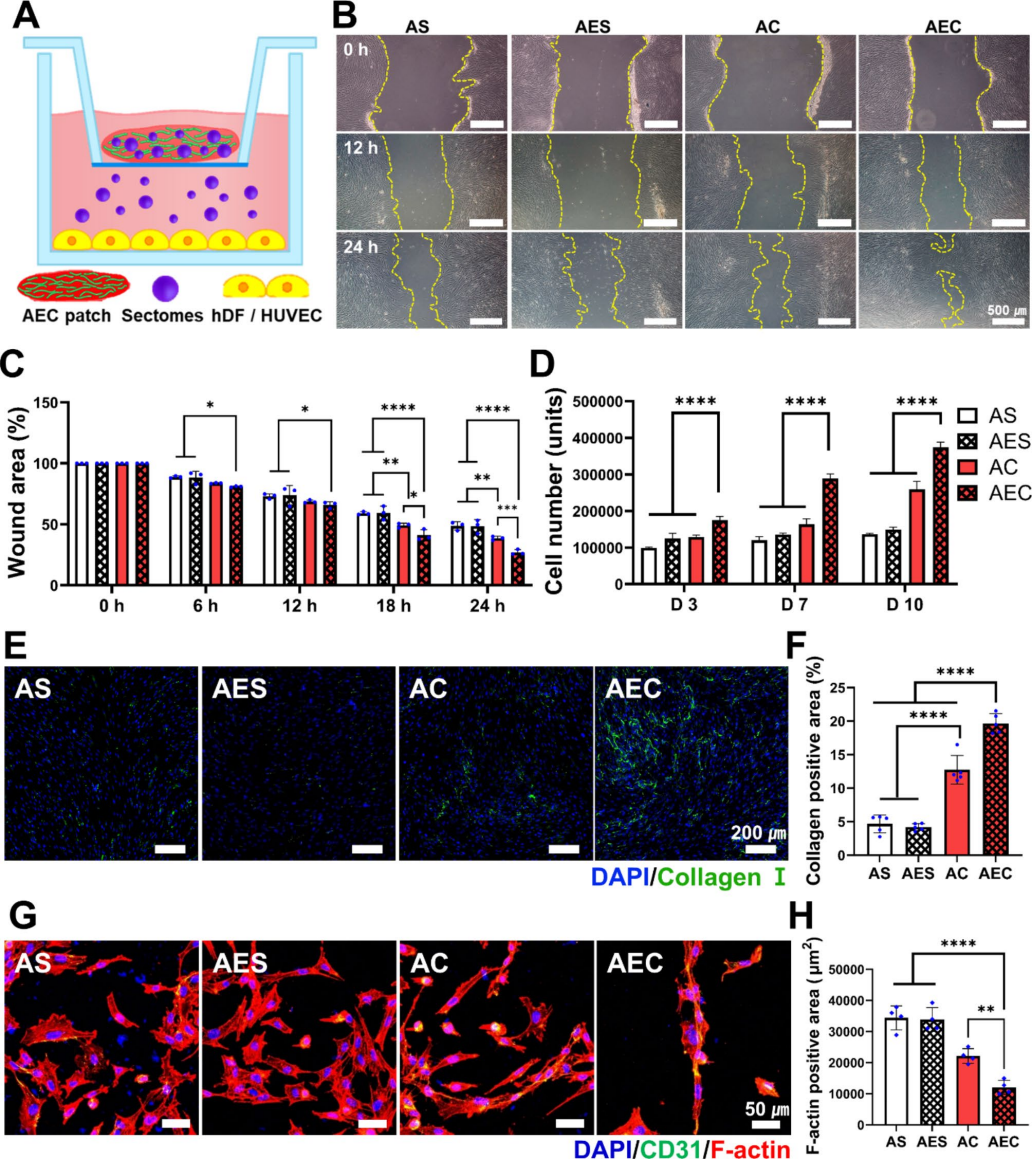
Assessment of biological activity of AEC patch in vitro. (**A**) Schematic of AEC patch-contained secretomes transport to the cells using a transwell setting. (**B**) Gross observation of hSFB scratch wound closure with time. The yellow line indicates the uncovered wound area with the cells. Scale bar is 500 μm. (**C**) Quantification of the wound closure rate at different time points for 24 h. (**D**) Cell proliferation of hSFB when treated with each patch group as measured by CCK-8 assay for up to 10 days. (**E**) Immunofluorescence staining of collagen type I (green) as synthesized by hSFB, along with DAPI staining (blue). (**F**) Quantification of collagen I positive area. (**G**) Immunofluorescence staining of HUVEC (blue: DAPI, green: CD31, red: F-actin). (**H**) Quantitative analysis of positively stained area of F-actin. Statistically significant difference (*p < 0.05, **p < 0.01, ***p < 0.001, ****p < 0.0001)

In order to investigate the biological effects of the secretomes released from the patches, we harnessed a transwell setting (Fig. 3A) and examined wound recovery using a scratch model of hSFB. Once each patch was positioned as illustrated in Fig. 3A, the wound area (%) as marked in yellow line was monitored for 24 h (Fig. 3B) and quantitatively analyzed (Fig. 3C). The wounds treated with the secretomes contained patches (AC and AEC) showed faster wound closure than the other groups. In particular, the AEC group disclosed notably better wound closure and the difference was statistically significant over the others. For the cell proliferation, when the number of cells was measured using the CCK assay, parallel to the result of wound closure, the AEC group revealed the highest proliferation activity all the times and the difference was also significant (Fig. 3D). Moreover, as the collagen deposition of hSFB was quantitatively evaluated by positively stained area of collagen I (Fig. 3E), we noticed that collagen synthesis markedly increased in the secretomes treated groups (AC, AEC) compared to the others (AS, AES) (Fig. 3F). More specifically, AEC patch exhibited the highest collagen deposition (19.66 ± 1.5%), followed by AC treated one (12.73 ± 2.1%), AES (4.67 ± 1.3%) and AS group (4.19 ± 0.5%). In addition, analysis of the angiogenic activity of the secretomes with HUVEC revealed that the AEC-treated group showed self-assembled, elongated cell morphology, as confirmed by F-actin and DAPI (Fig. 3G). In addition, CD31, a vascular differentiation marker was also identified in the cells as treated with the two secretome groups (AC and AEC). Quantitatively analyzed via positively stained area, we found that the total cell area treated with AEC (12,053 ± 2254 µm^2^) was significantly lower than that of AS (34,387 ± 3854 μm ^2^), AES (33,840 ± 3858 μm ^2^), and AC (22,091 ± 2372 μm ^2^) (Fig. 3H). Moreover, keratinocyte migration assay showed that secretomes-released from AEC patch was very effective in inducing keratinocyte migration (Fig. S2). We found significantly better keratinocytes migration when treated with the secretomes containing groups (AC, AEC) (Fig. S2B). Quantitative evaluation via the number of cells made the difference more clear, disclosing the most effectiveness with AEC patch (34.6 ± 1), followed by AC (22.1 ± 1), AS (11.7 ± 1) and AES (11.1 ± 1) (Fig. S2C).

### AEC patch treatment to murine full-thickness skin wound model

**Fig. 4:**
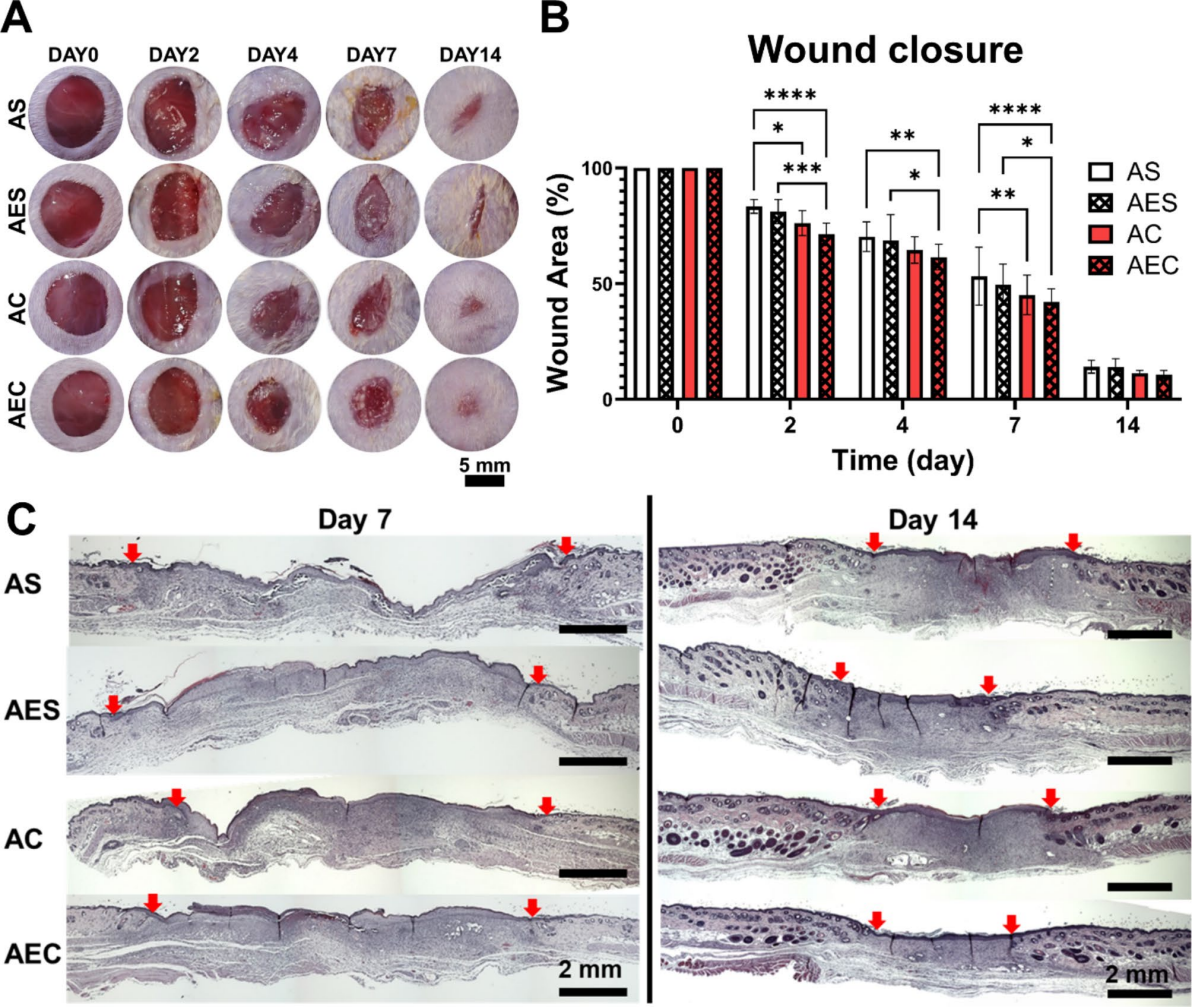
Observation of wound closure and histological analysis of wound tissues via. (**A**) Representative images of full-thickness skin wounds as administered by four different patch groups (AS, AES, AC, and AEC) at 0, 2, 4, 7, and 14 day post-treatment. Scale bar is 8 mm. (**B**) quantitative measurement of wound closure rate (%) for up to 14 days. (**C**) Representative images of regenerated wound tissues for each patch group on day 7 and 14 as stained by H&E. Red arrows indicate the wound edges. Scale bar is 2 mm. Statistically significant difference (*p < 0.05, **p < 0.01, ***p < 0.001, ****p < 0.0001)

We extensively investigated the therapeutic effect of alginate hydrogel patches using a full-thickness wound model (8 mm), administered with four different patch groups (AS, AES, AC, and AEC). The patches were easily removed from the wound sites when replaced every 2–3 day, where they were not absorbed or stuck to the wounds (Fig. S3A). As an in vivo safety assessment, we were unable to see any abnormal responses of subcutaneously transplanted AEC patch in gross observation (Fig. S3B). We also noticed no sign of adverse tissue reactions around the transplanted patch via H&E staining (Fig. S3C). The representative images of wound closure showed that the two secretome-treated groups (AC, AEC) had faster wound closure over time than those without secretomes (Fig. 4A). Quantitatively assessed, we noticed that the secretome groups healed wounds significantly better at early time point (Fig. 4B). Moreover, when we examined the remaining wound area as indicated with red arrows in the H&E staining (Fig. 4C), the result disclosed that on day 7 and 14, the secretomes-treated group (AC and AEC) presented significantly better wound recovery than the other groups.

### Advanced cell recruitment and neovascularization at early stage

**Fig. 5:**
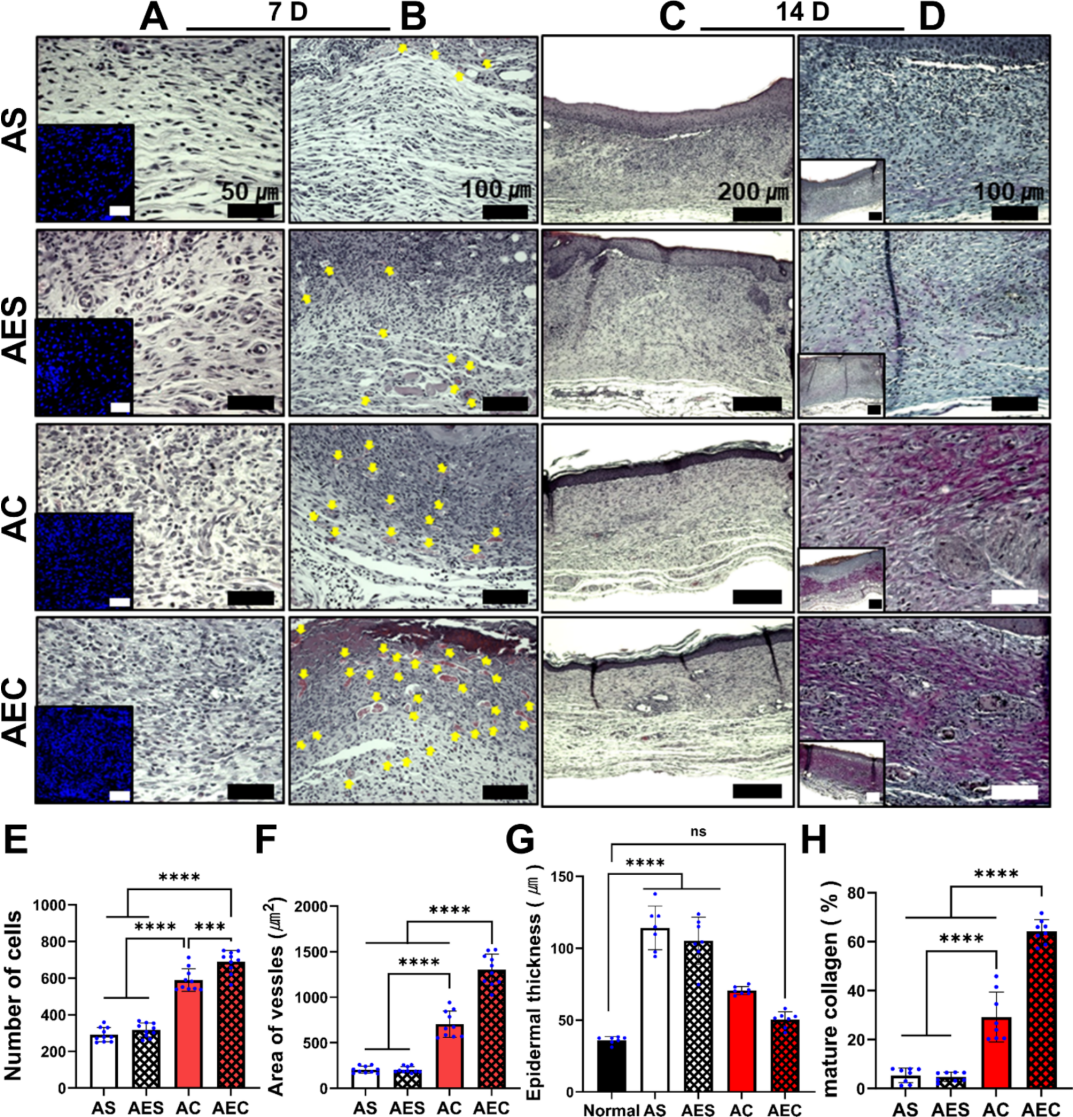
Histological analysis of the regenerated wound tissues on day 7 and 14. (**A**) High magnification images of the regenerated dermis stained by H&E and DAPI staining (inset) on day 7. Scale bar is 50 μm. (**B**) Medium magnification images of H&E-stained tissue samples. The yellow triangles suggest the neovessels in the dermis region. Scale bar is 100 μm. (**C**) Low magnification images of H&E samples on day 14. Scale bar is 200 μm. (**D**) High and lower magnification in the regenerated dermis as assessed by Herovici staining. Blue and purple color indicated young and mature collagen fibers, respectively. Scale bar is 100 μm. (**E**) Quantification of cellular recruitment in the dermis area on day 7. (**F**) Quantitative evaluation of the average neovessel size on day 7. (**G**) Measurement of the epidermis thickness and comparison with normal skin epidermis on day 14. (**H**) Quantitative analysis of mature collagen deposition in the regenerated dermis on day 14. Statistically significant difference (***p < 0.001, ****p < 0.0001)

The secretomes delivered by the AEC patch also promoted both cell recruitment and neovascularization at early stage of wound healing. On day 7, when treated with either AC or AEC patch, we observed active cell recruitment in the dermis region, with highly populated cells as assessed by the microscopic images of H&E and nucleic staining of DAPI (insets) (Fig. 5A). Quantitatively analyzed, the secretomes containing patch groups disclosed significantly higher cell numbers (AC: 589 ± 61.5 cells, AEC: 690 ± 60.4 cells) than the others (AS: 291 ± 38.8 cells, AES: 316.7 ± 38 cells) (Fig. 5E). It was notable that the difference between AC and AEC was also significant. Moreover, the neovessels (yellow arrows) in the dermis were detected in larger quantity from the histological images when treated with AC and AEC, respectively (Fig. 5B). Likewise, the AEC patch showed significantly better therapeutic indication, as supported by quantitative evaluation of the average neovessel area (AS: 204.3 ± 37 µm^2^, AES: 203.7 ± 35 µm^2^, AC: 705.2 ± 150 µm^2^, AEC: 1303 ± 173 µm^2^) (Fig. 5F).

### Recovery of normal epidermis thickness and mature collagen deposition

Secretomes delivered by the AEC patch contributed to the restoration of normal epidermis thickness and mature collagen deposition at later stage. H&E staining demonstrated a completely epithelialized layer with a normal epidermis layer at 14 day, when treated with the AEC patch (Fig. 5C). The quantitative assessment of the regenerated epidermis thickness at 14 day suggested no significant difference between AEC-treated wounds (50 ± 5.6 μm) and normal skin tissue (36 ± 2.5 μm) (Fig. 5G). Moreover, Herovici staining, which can differentiate between young (blue) and mature (red/purple) collagen showed that the secretome-treated groups (AC and AEC) had higher level of mature collagen deposition as appeared in purple color (Fig. 5D). The percentage of mature collagen was 64.2 ± 4.8% for AEC, followed by the AC group (29.2 ± 10.1%) (Fig. 5H). The AS (5.4 ± 3%) and AES (4.8 ± 1.9%) groups were very poor in collagen maturation.

### Accelerated keratinocyte migration and maturation of neovessels

**Fig. 6:**
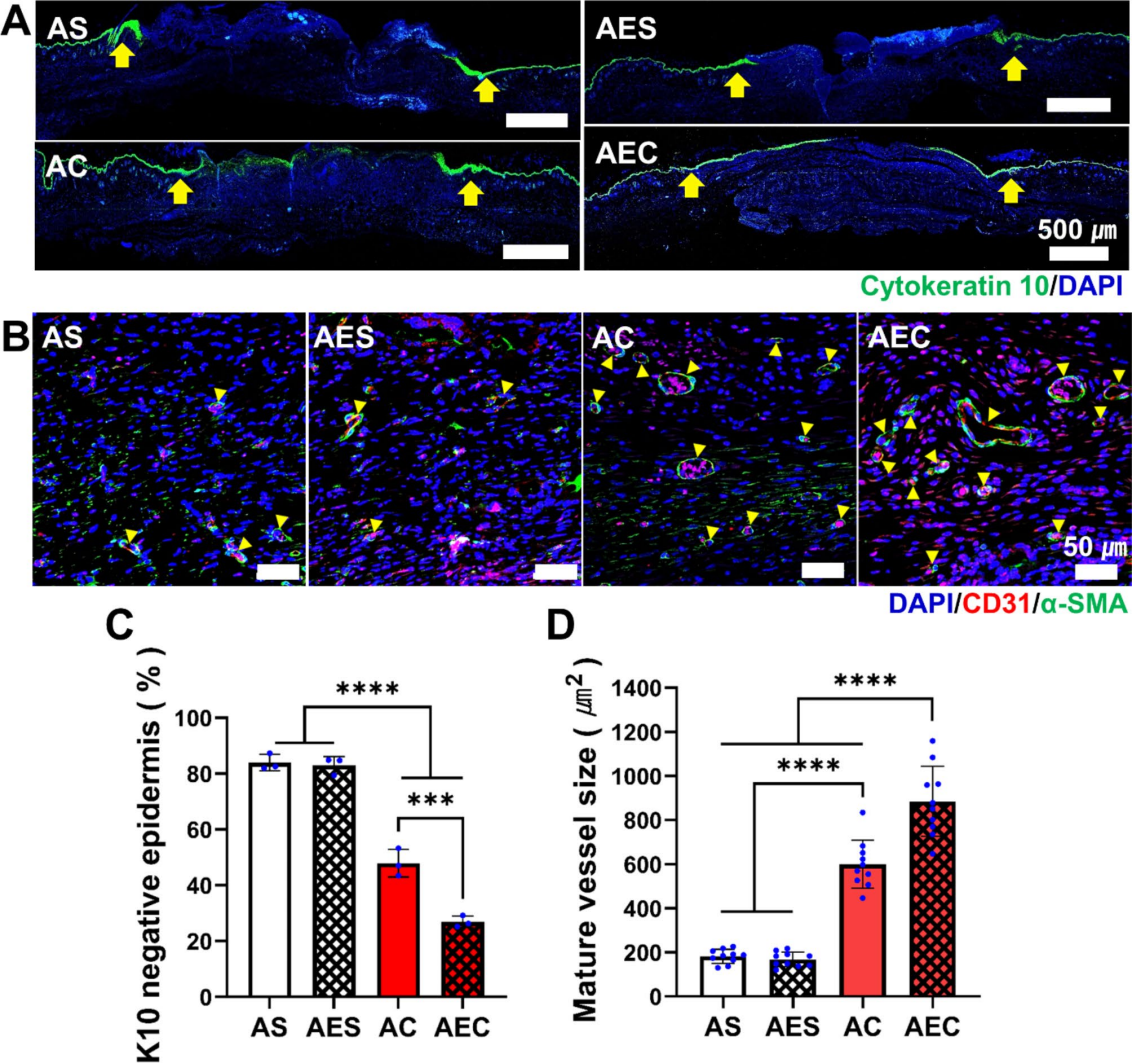
In-depth analysis of the wound tissues via immunofluorescence staining. (**A**) Representative immunofluorescence images of wound tissues on day 7 (green: cytokine10 (K10), blue: DAPI). The yellow arrow indicates the wound edges. Scale bar is 500 μm. (**B**) Representative images of mature neovessels as assessed via co-immunofluorescence of α-SMA (green) and CD31 (red), along with DAPI staining. The yellow triangles denote mature vessels. Scale bar is 50 μm. (**D**) Quantitative analysis of the epidermis area (%), which was K10 negative on day 7. (**E**) Quantitative measurement of average mature vessel size on day 14. Statistically significant difference (***p < 0.001, ****p < 0.0001)

For more in-depth analysis, epithelialization of wound site through keratinocyte migration was further investigated using IF images of cytokeratin 10 (K10), a keratinocyte marker (Fig. 6A). Although a complete epithelialization was not confirmed at 7 day in all groups, the secretomes-treated groups (AC, AEC) disclosed a wider distribution of K10 positive area in the epidermis than the other groups (AS, AES). Quantitatively assessed, the percentage of K10 negative area relative to the original wound length was significantly lower in the AEC (26.9 ± 2%) and AC (47.9 ± 5%) treatment groups, while AS (84 ± 2.9%) and AES (83 ± 3.1%) showed a significantly larger percentage, which meant the poor recovery of epidermis (Fig. 6C). On day 14, the mature blood vessel-like structure was further characterized via co-immunofluorescence of the CD31 marker (red) for endothelial cells (ECs), which was surrounded by α-SMA (green) for smooth muscle cells (SMCs), as indicated by the yellow arrows (Fig. 6B). The presence of the mature vessel-like structure was more prevalent in the AC and AEC group. The quantitative evaluation of the mature vessels area presented a significant difference among the test groups: AEC (982 ± 72.2 µm^2^), AC (561.4 ± 48.3 µm^2^), AES (151 ± 9.7 µm^2^), and AS (163.1 ± 17.4 µm^2^) (Fig. 6D). Collectively, these results clearly supported that the secretomes delivered by our AEC patch could accelerate the re-epithelialization at an early stage and the maturation of neovessels at later time point during the wound healing process.

### Assessment of the myofibroblast during wound healing

**Fig. 7:**
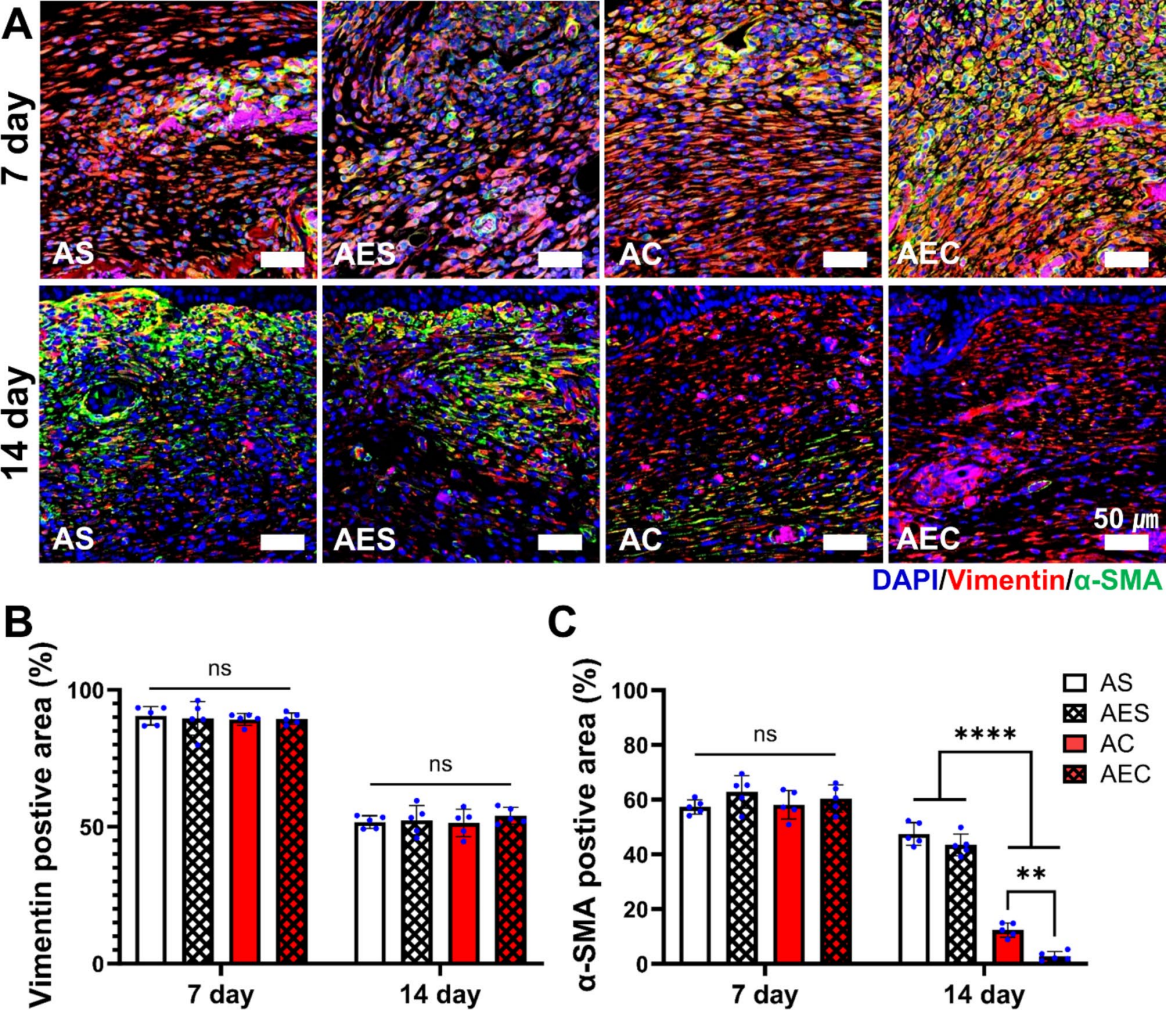
In-depth analysis of myofibrobalsts activation during wound healing process. (**A**) Co-immunofluorescence images of the regenerated dermis on day 7 and 14 (green: α-SMA, red: vimentin, blue: DAPI). Scale bar is 50 μm. (**B**) Quantitative assessment of vimentin positive area (%) in the dermis on day 7 and 14. (**C**) Quantitative assessment of α-SMA positive area (%) in the dermis on day 7 and 14. Statistically significant difference (**p < 0.01, ****p < 0.0001)

The balance between fibroblast and myofibroblast is crucial in determining the quality of wound regeneration. During wound repair, activation of myofibroblasts play many important roles, including the production of new ECM. To this end, we were interested in the role of secretomes regarding the activation/deactivation of myofibroblasts. For this, wound tissue sections on day 7 and 14 were subjected to IF staining using α-SMA (green) for activated myofibroblasts and vimentin (red) to identify all fibroblasts, including myofibroblasts (Fig. 7A). We noticed that myofibroblasts populations were co-stained with α-SMA and vimentin, which were observed in all the groups on day 7. However, the α-SMA (+) staining was more dense and stronger in the AEC-treated wounds than the others. Interestingly, the fibroblasts population, positive only with vimentin became dominant at 14 day in the secretome groups (AC and AEC). Instead, those activated myofibroblasts labeled only with α-SMA were highly populated in the AS and AES treated wounds on day 14. In the quantitative evaluation of vimentin (+) area, there was no significant difference among the four test groups at 7 (89.7 ± 0.6%) and 14 (52.4 ± 1.2%) day, respectively (Fig. 7B). The quantitative assessment of α-SMA (+) area revealed that there was no significant difference among the test groups at 7 day (60 ± 2.5%) (Fig. 7C). Interestingly, we saw a dramatic shift of cell population on day 14: AEC (2.8 ± 1.7%) and AC (12.4 ± 2.6%) treated group disclosed significantly decreased level of α-SMA (+) area. Both AS (47.5 ± 4.2%) and AES (43.5 ± 4%) still remained high in activated myofibroblasts.

## Discussion

In this study, we successfully developed the AEC patch through several processes. Our patch is a very thin membrane in a dry state but it can absorb CCM quickly to become a hydrogel patch, which can provide a moisture environment for the wounds. It was easily removed upon changing the dressing every 2–3 day (Fig. S3A). Any sign of severe inflammation or adverse tissue reactions was undetectable in the full-thickness wounds or subcutaneous model (Fig. S3B, C), suggesting in vivo safety of our patch. Current results supported that our AEC patch was very effective in loading and delivering various bioactive molecules in the secretomes. The therapeutic effects of such factors release were also clearly demonstrated using in vitro cells and full-thickness wound model. A key point in our patch system is the inclusion of ECM. We postulated that our ECM may function in a similar way as it does in vivo, where it can secure the GF and cytokines. In fact, there was a huge difference between AC (without ECM) and AEC in terms of not only the holding efficiency but wound healing capability. Our decellularized ECM includes collagen, laminin, and fibronectin, each of which has unique binding sites for specific GFs [27]. The ECM in the native tissues plays a regulatory role in the storage and transmission of GFs through specific binding mechanism [28, 29]. For example, fibronectin can bind HGF through receptor and integrin and has a domain that forms a complex with VEGF [24]. Laminin also has multiple isoforms that can bind multiple GFs, such as VEGF, PDGF, FGF, and IGF through heparin-binding domains [26]. Diverse types of collagens and vitronectin are also involved in the similar function [27, 30]. Early works harnessed one of the ECM components selectively or manipulated biomaterials to improve the efficacy of GFs delivery [28, 31, 32]. Our cell-derived, decellularized ECM is a complex of diverse ECM components, which retains plenty of GFs-binding sites intrinsically and proved high efficiency of the CCM-contained GFs delivery. The direct comparison of representative bioactive factors (VEGF, HGF, IGFBP, IL-6 and IL-8) disclosed a statistically significant difference, where the AEC patch could hold significantly larger amount of those factors over the AC patch, which was absent of ECM.

To incorporate such bioactive molecules as much as possible in our patch, the hMSCs-derived CM was subjected to further process of centrifugation to obtain the CCM, which was highly enriched secretomes in a significantly reduced volume. This process enabled our AEC patch to reserve high concentration of growth factors and cytokines. Such bioactive factors would play a crucial role in the wound healing. For example, VEGF, a representative angiogenic factor, is involved in granulation tissue formation and peripheral nerve regeneration [19]. The HGF would facilitate re-epithelialization, granulation tissue formation, and angiogenesis [33]. The insulin-like growth factor (IGF) activated by IGFBP is also known to promote cell proliferation and re-epithelialization [34, 35]. In addition, the inflammatory cytokines, IL-6 and IL-8 are closely related to re-epithelialization and keratinocyte migration [18]. The beneficial impact of those factors were conceivable during wound healing process and they may function either individually or collectively. Likewise, various biomaterials would incorporate specific GFs extrinsically as a main mediator of wound healing in many studies [36, 37].

When the biological effects of the AEC patch were assessed, the in vitro scratch assay and cell proliferation test indicated that the secretomes released out of the AEC patch could affect hSFB behaviors, significantly promoting cell migration and proliferation, both of which are crucial requirements for wound healing in vivo. Upon the fact that ECM production is critical in complete wound healing [3], active collagen deposition of skin fibroblasts suggested that hMSCs-derived secretomes were also effective in facilitating collagen synthesis. Collagen is a key ECM component in the skin tissue, which is mostly produced by fibroblasts. Both IL-6 and IGFBP, which were abundant in our secretome, are indirectly involved in collagen synthesis [38, 39]. Meanwhile, our results proved the role of angiogenic GFs (HGF, VEGF and IL-6) contained in the secretomes, as assessed by self-assembly of HUVECs in vitro. We also identified excellent wound healing indications in vivo by administering the AEC patch. During the early time period (7 day), wound closure was significantly accelerated when treated with the AEC patch. The regenerated wound tissues showed distinct skin layers (epidermis, dermis, and hypodermis) at 14 day and the thickness was similar to normal skin tissue (Fig. 5). Such therapeutic molecules in the secretomes definitely led to the regenerative effect in the wounds. During wound regeneration, cell recruitment and neovascularization are the typical examples of therapeutic mechanism. Since paracrine effects have been recognized as a working principle of stem cell therapy, our AEC patch may recapitulate a similar therapeutic mechanism as it effectively delivered MSCs-mediated secretomes into the wound site and led to advanced wound regeneration. Among the secretomes delivered by our AEC patch, HGF and VEGF are well-known factors involved in angiogenesis [19, 40]. The pro-inflammatory cytokines, IL-6, and IL-8 induce cell recruitment in the wound healing process. TGF-β and PDGF present in our secretomes (Fig. 1C) would also play a crucial role in stimulating vascular cells proliferation and neovessels maturation [19]. Angiogenesis is initiated by the assembly of HUVECs to form a tube and such neovessels are essential in the movement of nutrients, oxygen, and cells to the wound site [41]. In the remodeling phase, blood vessels maturation undergoes, where ECs were surrounded by SMCs for a tube formation [42]. Taken together, our results supported that the secretomes delivered by the AEC patch could enhance cell migration and proliferation, facilitate collagen production, and promote neovascularization in vivo. Interestingly, the difference of wound healing capability between the two secretomes groups (AEC vs. AC) was notable. We found that the AEC patch had significantly better therapeutic effect than the AC, even though they were initially loaded with the same amount of CCM. It was obvious that the ECM incorporated in the AEC patch made such difference. As confirmed by ELISA, AEC patch could hold significantly larger amount of VEGF, HGF, IL-6 and IL-8 than the AC one, even though they were processed in the same condition (Fig. 2I-M). These results strongly suggested the active role of our ECM, such as ECM-growth factors interactions and enhanced loading efficiency as a result, where those actions positively affected cellular activity in vitro and also tissue regeneration in vivo.

We also paid attention to the specific cell types, which were deeply involved in wound healing process (Figs. 6 and 7). First of all, epithelization is an important step in the proliferation phase of wound healing. Keratinocyte is an essential cell type that constitute the epidermis, where they are activated from the wound edge and migrated to the wound site through proliferation and differentiation [33]. Among the diverse secretomes contained in the AEC patch, HGF and IL-6 are known to be epithelialization-inducing factors [18, 19]. EGF and IGF are also recognized as the major factors inducing keratinocyte migration to wound sites [33]. The same effect was reproducible in our keratinocyte migration assay in vitro (Fig. S2). As a result, AEC patch-treated wounds were found most effective in forming the epithelialized layer (Fig. 6A). Meanwhile, myofibroblasts are another important cell type, where they not only induce wound contraction in the proliferation phase, but produce new ECM, and restore the wounds through communication with the neighboring cells [3]. They are also involved in granulation tissue development and in regeneration of the skin appendages, such as blood vessels and sweat glands. [43]. Once their duty was completed, they are supposed to be deactivated by apoptosis or differentiated into fibroblasts in the remodeling phase. Persistence of myofibroblasts activity would cause chronic inflammation and excessive scarring [44]. Interestingly, we noticed a sequential, dramatic shift of cell type in the AEC patch treated wounds, where myofibroblsts were prevalent at 7 day but later fibroblasts became dominant at 14 day (Fig. 7A). The difference compared to the other three groups was statistically significant (Fig. 7D). The primary reason we postulate is the contributions either individually or collectively of biologically active factors during the wound healing process. Among them, VEGF, IL-6, and IL-8 are the factors that can induce activation of myofibroblasts. On the contrary, EGF and basic fibroblast growth factor (bFGF) (Fig. 1C) are linked to the deactivation of myofibroblasts [45]. We think the activation/ deactivation mechanism of myofibroblasts via hMSCs-dereived secretomes requires further in-depth study. Other than soluble factors, deactivation of myofibroblasts is also affected by physical and mechanical environment of the surrounding tissue [46]. Such enhanced mature collagens deposition in the AEC-treated group (Fig. 5D, H) might have partly contributed to the deactivated myofibroblasts at later time point.

## Conclusions

In this study, we have successfully developed an alginate-based hydrogel patch (AEC), where it contains hMSCs-derived secretomes and human fibroblast-derived, decellularized matrix. We learned that the secretomes reserved diverse therapeutic factors, such as VEGF, HGF, IGFBPs, IL-6, and IL-8. Our AEC patch can effectively hold and deliver the secretomes into the wound site, where our ECM played a significant role in enriching those bioactive molecules in the patch. The AEC patch enhanced the cellular activity of dermal fibroblasts and endothelial cells in vitro and it also significantly improved wound healing quality in full-thickness skin wound model. Although the working mechanism of AEC is not fully defined at this time, we believe that our AEC patch could provide an environment for efficient secretomes reservation and release, due mainly to the presence of ECM that has multiple bind capacity for diverse growth factors and cytokines. This platform may be applied for other tissues regeneration using specific secretomes derived from target-oriented cell type. Collectively, our AEC patch demonstrated advanced wound tissue regeneration, where the secretomes could exert significant therapeutic effects either individually or collectively during the wound healing process.

### Electronic supplementary material

Below is the link to the electronic supplementary material.

Supplementary Material 1

## Data Availability

The datasets used and/or analyzed during the current study are available from the corresponding author on reasonable request.

## Abbreviations

AC: Alginate/CCM
AEC: Alginate/ECM/CCM
AES: Alginate/ECM/SFM
ALG: Alginate
AS: Alginate/SFM
bFGF: Basic fibroblast growth factor
BMP: Bone morphogenetic protein
BSA: Bovine serum albumin
CCK-8: Cell counting kit-8
CCM: Concentrated conditioned media
CM: Conditioned media
DAPI: 4, 6-diamidino-2-phenylindole
DMEM: Dulbecco’s modified Eagle’s medium
DW: Deionized water
EBM: Endothelial cell basal medium
ECM: Extracellular matrix
EGF: Epidermal growth factor
EGM: Endothelial cell growth medium
ELISA: Enzyme-linked immunosorbent assay
FBS: Fetal bovine serum
FGF: Fibroblast growth factor
GF: Growth factors
H&E: Hematoxylin and eosin
HGF: Hepatocyte growth factor
hMSC: Human mesenchymal stem cells
hSFB: Human skin fibroblasts
HUVEC: Human umbilical vein endothelial cell
IF: Immunofluorescence
IGF: Insulin-like growth factor
IGFBP: Insulin-like growth factor binding protein
IL-1: Interleukin-1
IL-6: Interleukin-6
IL-8: Interleukin-8
KBM: Keratinocyte basal medium
KGM: Keratinocyte growth medium
MSC: Mesenchymal stem cells
P/S: Penicillin and streptomycin
PBS: Phosphate buffered saline
PDGF: Platelet-derived growth factor
RT: Room temperature
SFM: Serum-free medium
TCP: Tissue culture plate
TGF-β: Transforming growth factor-β
TNF-α: Tumor necrosis factor-α
TRITC: Tetramethylrhodamine
VEGF: Vascular endothelial growth factor
α-SMA: alpha smooth muscle actin
