# Supplementary material for: Mesenchymal stem cell-derived secretomes-enriched alginate/ extracellular matrix hydrogel patch accelerates skin wound healing
Source: Biomater Res. 2023 Oct 31;27:107. doi: 10.1186/s40824-023-00446-y (PMC10617187; doi:10.1186/s40824-023-00446-y)
Supplement: Supplementary file 1 — Supplementary Material 1 [file 40824_2023_446_MOESM1_ESM.docx]

**Supplementary Data**

**Mesenchymal stem cell-derived secretomes-enriched extracellular matrix/alginate hydrogel patch accelerates skin wound healing**

Jae Won Kwon^1,2^, Cininta Savitri^1^, Byoungha An^1,2^, Seung Won Yang^1,2^, Kwideok Park^1,2, *^

^1^Center for Biomaterials, Korea Institute of Science and Technology (KIST), Seoul 02792, Republic of Korea

^2^Division of Bio-Medical Science and Technology, University of Science and Technology (UST), Daejeon, 34113, Republic of Korea

Jae Won Kwon: Republic of Korea, kwonjw95@kist.re.kr

Cininta Savitri: Republic of Korea, [casavitri@kist.re.kr](mailto:casavitri@kist.re.kr)

Byoungha An: Republic of Korea, abh0722@kist.re.kr

Seung Won Yang: Republic of Korea, h20512@kist.re.kr

*Corresponding author: Kwideok Park

Center for Biomaterials, Korea Institute of Science and Technology (KIST), Seoul 02792, Republic of Korea

E-mail address: kpark@kist.re.kr

Tel: +82-2-958-5288

Fax: +82-2-958-5308

**Supplementary Figure 1**


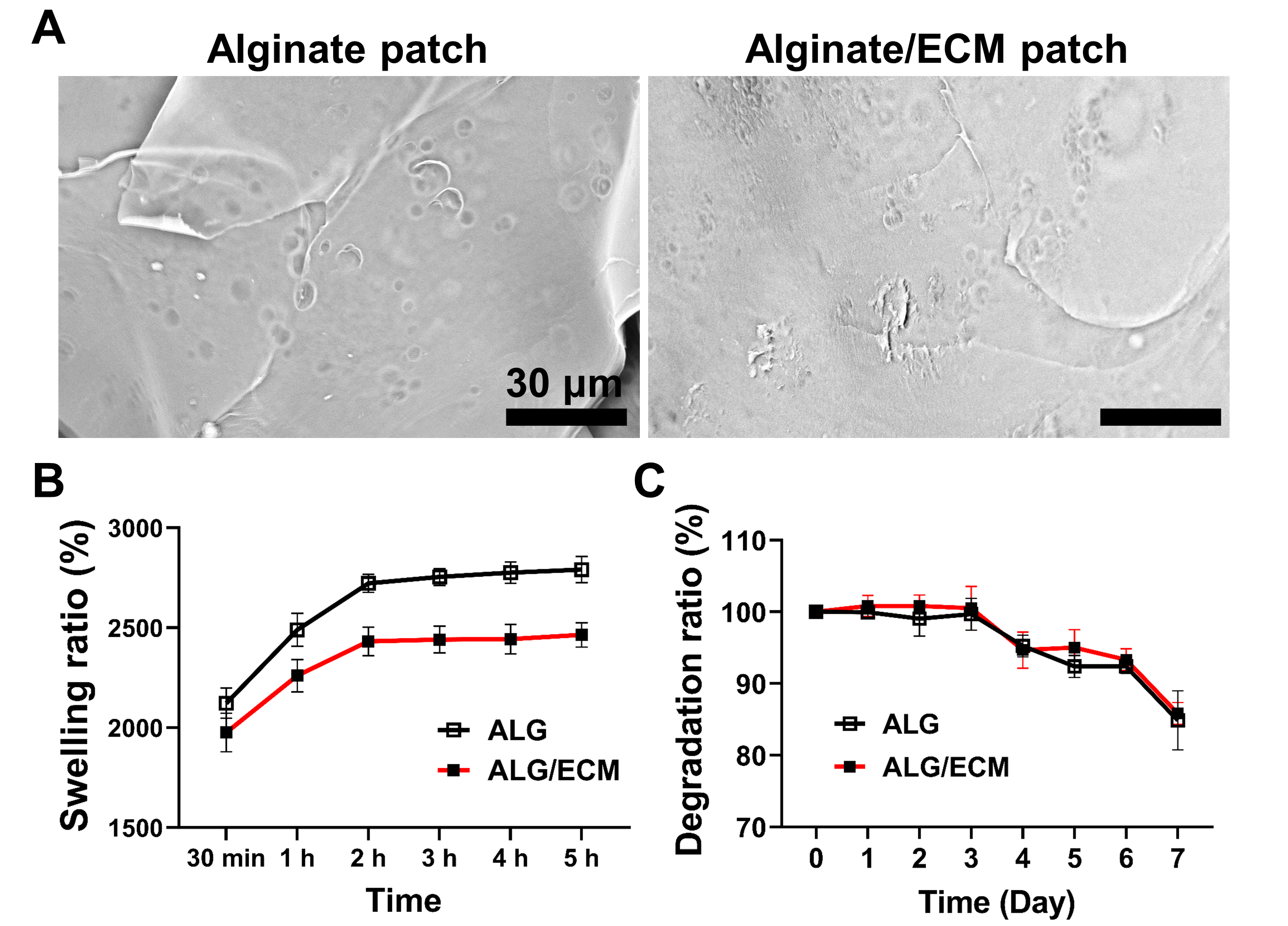


**Fig. S1.** Characterization of alginate and alginate/ECM patch. (A) Surface morphology of each patch (left: alginate, right: alginate/ECM) as assessed via SEM. Scale bar is 30 µm. (B) Swelling ratio (%) of each patch in normal saline: ALG (alginate) and ALG/ECM (alginate/ECM). (C) Degradation ratio (%) of each patch for up to 7 days *in vitro*.

**
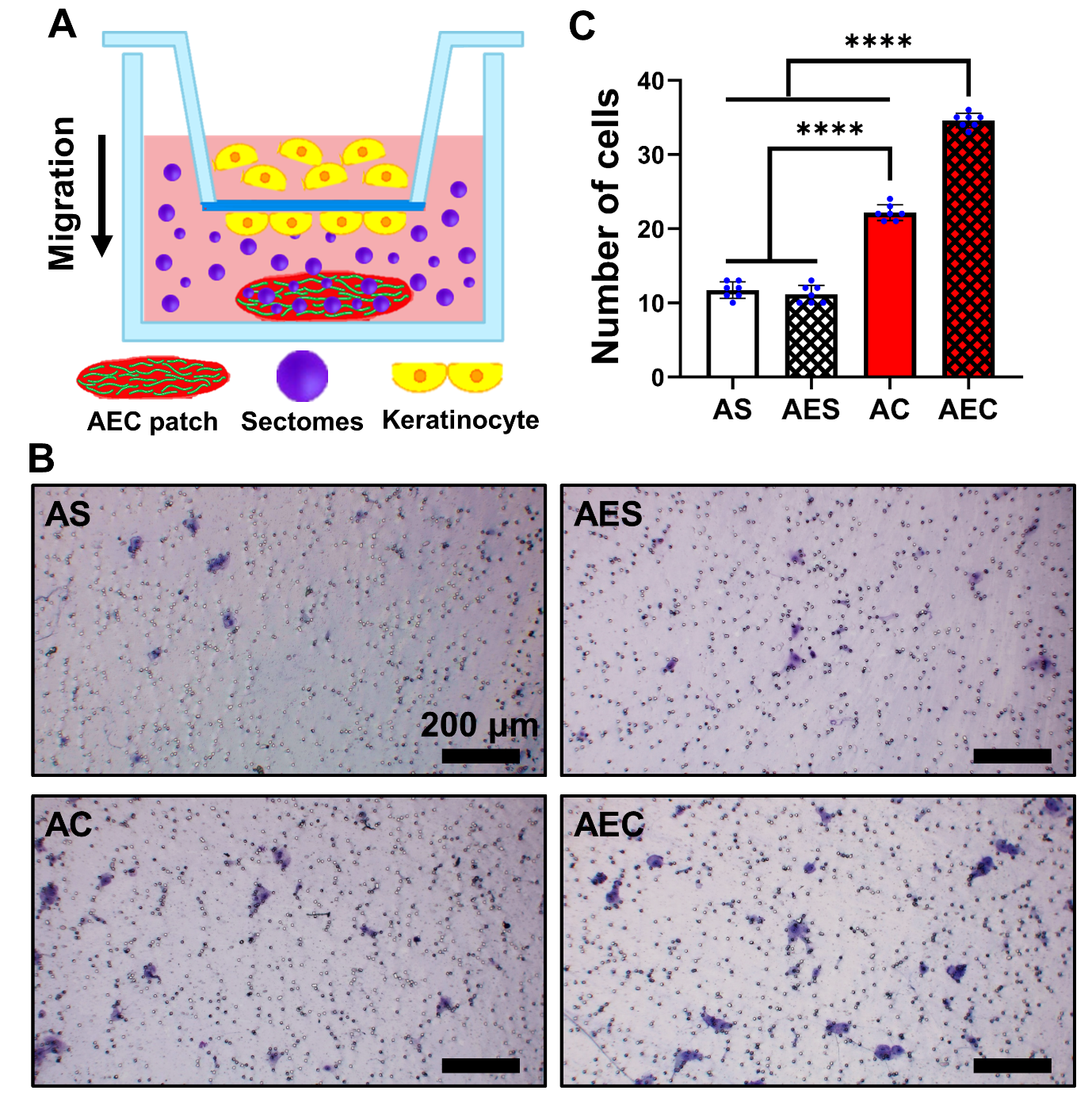
Supplementary Figure 2**

**Fig. S2.** Keratinocyte migration assay. (A) Schematic of transwell migration assay with AEC patch-contained secretomes. (B) Representative images of the migrated keratinocytes (purple) as assessed via crystal violet staining. Scale bar is 200 µm. (C) Quantitative evaluation of keratinocyte migration by counting the number of cells. Statistically significant difference (*****p*< 0.0001).

**Supplementary Figure 3**


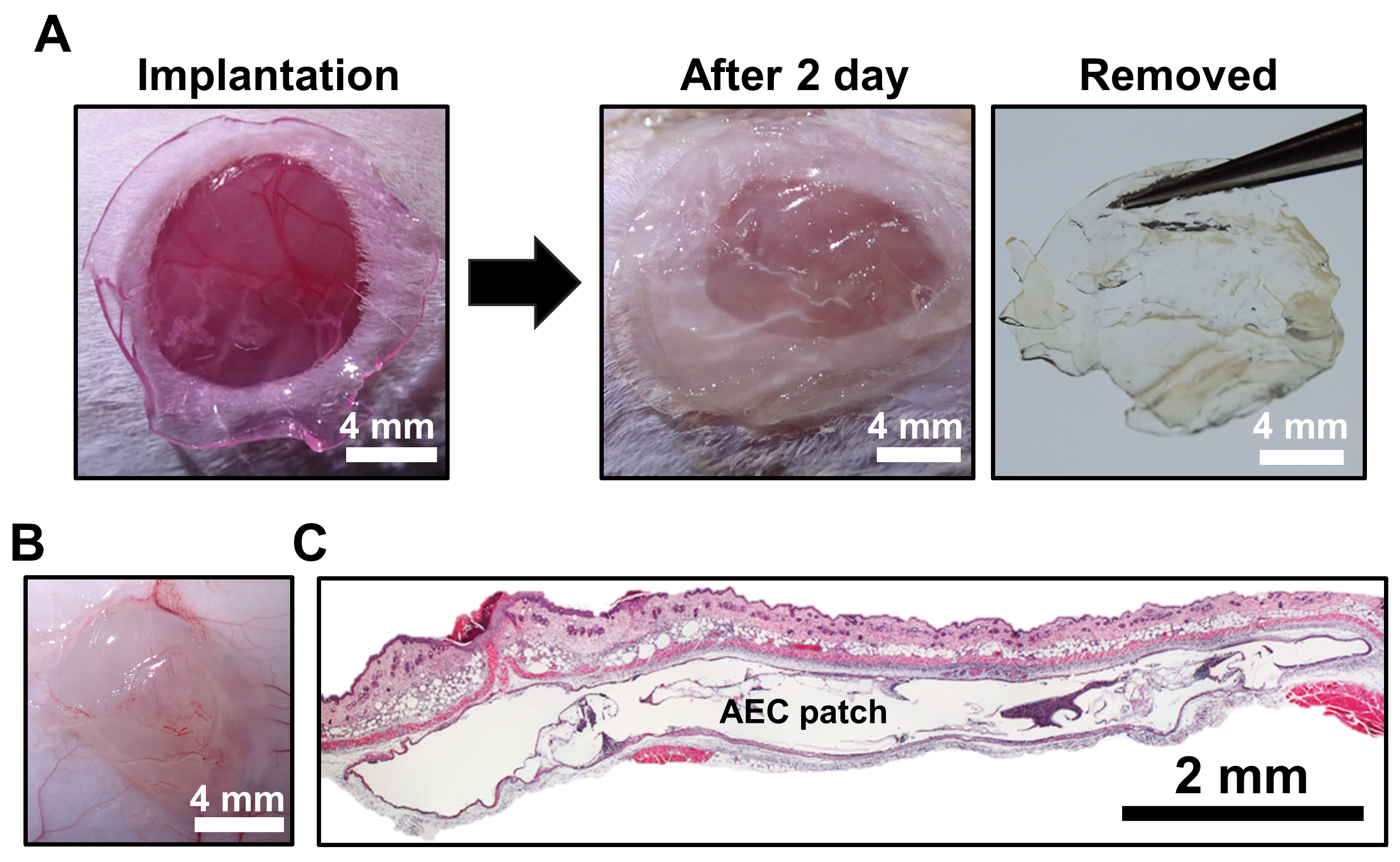


**Fig. S3.** Observation of AEC patch transplanted in the full-thickness wounds *in vivo*. (A) Gross images of AEC patch-transplanted wounds (left: right after implantation, middle: 2 day post-implantation, right: AEC patch removed from the wound). Scale bar is 4 mm. (B) Gross image of subcutaneously transplanted AEC patch. (C) Representative images of the transplanted AEC patch on day 3 as assessed by H&E staining. Scale bar is 2 mm.
